# Strychnia in Anæsthesia

**Published:** 1866-09

**Authors:** Samuel C. Chew

**Affiliations:** Professor of Materia Medica and Therapeutics in the University of Maryland


*Strychnia in Anæsthesia.* By SAMUEL C. CHEW, M. D., Professor of Materia Medica and Therapeutics in the University of Maryland.

The influence of strychnia, as a spinal stimulant, is chiefly exerted, as is well known, upon the anterior column of the cord, and through the motor nerves originating in that portion of the spinal axis, it gives rise to muscular contractions.

It was first employed as a remedy for paralysis, more than fifty years ago, by Fouquier, who pointed out with great accuracy the peculiar indications for the use of strychnia, and whose observations have been abundantly corroborated by later experimentation.

According to Brown-Sequard, the action of strychnia consists in an increased nutrition of the spinal marrow, in consequence of which a larger amount of nervous force is generated and conducted along the nerves to the muscles; and this conclusion agrees with that arrived at by Kœlliker, who has proved that the drugs acts upon the spinal marrow alone, and not directly upon the nerves themselves, nor upon the muscles to which the nerves are distributed. This is rendered evident by the simultaneous contraction and relaxation of many muscles, which can be occasioned only by an influence acting upon their common source of innervation; and that this source is the cord, independently of its connection with the brain, is proved by the fact, that in the midst of the tetanic convulsions, the central functions are often found to be entirely unaffected; and by the further fact, that in some of the lower animals, if the cord be severed from the brain, the same phenomena are caused by strychnia as when the connection is unbroken.

In its influence upon the cord, strychnia, as just stated, appears to affect principally the motor tract. In many cases of paralysis of motion, in which sensation is unimpaired, power is rapidly restored to the paralyzed muscles by its use, and when a poisonous dose of strychnia has been taken by a person in health, though the spasmodic contractions of the muscles may be of the most violent character, yet very generally no hyperæsthesia or exaltation of sensibility is produced. It often happens, indeed, that little or no pain is felt, even in the midst of the convulsions, the stiffness and soreness of the muscles that are subsequently experienced being due to muscular exhaustion, as a consequence of excessive action.

Though the effects of strychnia are exerted chiefly upon the motor tract of the cord, it must not however be regarded as limited in its action exclusively to this region. After it has been administered in small medicinal doses for some days, patients occasionally experience a sense of formication, tingling or itching in various parts of the body, resembling what is felt, when the foot is said to be asleep. This shows that it exerts a certain degree of influence over the sensibility of the nervous centres, stimulating the function of sensation to some extent, but much less than that of motion. In the treatment of paraplegic cases, in which sensation and motion are both abolished, when strychnia is indicated, its use is found to be followed by a restoration of sensibility, as well as of the power of motion. And still further, in some rare cases, it appears to act principally, if not exclusively, upon the posterior column, when the function of sensation is impaired, without the existence of any corresponding disturbance in that motion. M. Petrequin reports instances, in which he had used it successfully in the treatment of anæsthesia; and cases of amaurosis and impairment or abolition of the senses of taste, smell and hearing furnish illustrations of its beneficial effects upon sensory paralysis.

The following case is of interest, as showing a very marked action of strychnia upon a paralysis of sensation, alone affecting an extensive surface:

J. B., a native of Pennsylvania, aged 52 years, was admitted into the Baltimore Infirmary, October 8th. He had suffered from cough, more or less constantly, for more than two years, and had lost flesh and strength. Upon his admission, the signs of a small cavity in the right lung were discovered. He was put upon the use of cod liver oil, and under its influence the pulmonary trouble underwent a marked alleviation.

About a month before his entrance into the Infirmary, the patient became aware of a diminution of sensibility in the left foot; this was for a time so slight that he made no mention of it to the attending physician. Gradually, however, the anæsthesia extended upwards, from the foot to the knee, and on taking charge of the Infirmary, in March, I found that sensibility was entirely abolished in the foot and lower part of the leg, and very much impaired for the distance of three or four inches above the knee. In all other parts of the body, it was unaltered. The power of motion appeared to be affected only secondarily, as a consequence of the loss of sensation. The patient could move his toes, and had control over all the muscles of the limb, but not feeling the contact of his foot with the ground, his gait was unsteady, and unless his eye was kept fixed upon the foot which had lost sensation, he was liable to fall down.

The method suggested by Weber, for ascertaining the degree of cutaneous insensibility, which consists in determining how closely the points of a pair of compasses may be approximated on the skin, and yet be distinguished as two points, was entirely inapplicable to the lower part of the limb, as sensibility was utterly abolished. A much rougher test was necessary, and accordingly I made, with a bistoury, two incisions through the skin, one just above the outer malleolus, the other just below the knee, without causing the patient to manifest the least sign of pain; and the same insensibility was shown on applying a lighted match to an intermediate point.

Thinking it a favorable case for testing the effect of strychnia, upon sensory paralysis, apparently uncomplicated with disturbance of motor power, I directed the patient to have the 48th part of a grain of strychnia in solution three times a day, and had the affected limb rubbed at the same intervals, with a liniment composed of equal parts of Tr. Nucis Vomic. and Tr. Arnicæ.

In about a week there was a very decided increase of sensibility in the upper part of the limb, and some evidence of a return of feeling in the foot, which in the course of two or three weeks more became very perceptible.

The dose of strychnia was now increased to the 24th part of a grain, and continued in this amount for several weeks. The degree of sensibility increased steadily and rapidly, until by the 1st of June there was no apparent difference in this respect, between the two limbs—the application of irritants affecting the one that had previously been anæsthetic, fully as much as the other.

The patient was now no longer liable to fall down when his eye was averted from his foot. He could walk without inconvenience, for a considerable distance, and was able to go up stairs without using his cane.

The return of sensibility was fairly attributable to the use of strychnia, inasmuch as the anæsthesia, which had been absolute, began to abate almost immediately after the first administration of the drug, and constantly diminished until the normal function was entirely restored; the ability to walk being altogether dependent upon the return of sensory power.

The pathology of this and similar cases is interesting, and yet very obscure. The dilemma of an eccentric or a centric lesion is presented. If the paralysis be supposed to depend upon an eccentric lesion affecting a nerve in its course, such as mechanical pressure, or a diseased condition of its fibres, how is it to be explained, that sensation is affected independent of motion? Can we suppose the pressure or the disease to act upon the sensory filaments alone, and not upon the motor also, when the two roots of the spinal nerves, after emerging from the cal, become so blended together, that their respective filaments can no longer be distinguishable ?

On the other hand, if the lesion be centric—seated, that is, in the posterior column of the cord, or in the posterior root, before it has become united with the anterior—why is it that the seat of sensation in the cord was affected by impressions transmitted along the sensory nerves from the upper part of the limb, which never lost sensibility, and not at all affected by impressions which came from its lower parts ?

In the present state of our knowledge of the nervous system, no satisfactory explanation of these phenomena can be offered, and we are obliged to accept them as ultimate facts.—*Richmond Med. Journal.*

